# Effects of Continuous Prenatal Low Dose Rate Irradiation on Neurobehavior, Hippocampal Cellularity, Messenger RNA and MicroRNA Expression on B6C3F1 Mice

**DOI:** 10.3390/cells13171423

**Published:** 2024-08-26

**Authors:** Feng Ru Tang, Ignacia Braga Tanaka, Hong Wang, Salihah Lau, Satoshi Tanaka, Amanda Tan, Daisaku Takai, Akiko Abe

**Affiliations:** 1 Radiation Physiology Lab, Singapore Nuclear Research and Safety Initiative, National University of Singapore, Singapore 118415, Singapore; snrwh@nus.edu.sg (H.W.); salihahl@nus.edu.sg (S.L.); amanda97@nus.edu.sg (A.T.); 2 Department of Radiobiology, Institute for Environmental Sciences, 2-121 Hacchazawa, Takahoko, Rokkasho, Aomori 039-3213, Japan; tanakaib@ies.or.jp (I.B.T.III); tanakas@ies.or.jp (S.T.); 3 Tritium Research Center, Institute for Environmental Sciences, 2-121 Hacchazawa, Takahoko, Rokkasho, Aomori 039-3213, Japan; dtakai@ies.or.jp; 4 JAC Co., Ltd., 1-2-7 Higashiyama, Meguro, Tokyo 153-0043, Japan; a.todate@jac-co.co.jp

**Keywords:** prenatal, low dose rate, chronic radiation exposure, brain effect

## Abstract

Epidemiological, experimental, and ecological data have indicated the controversial effect of in utero chronic low dose rate (<6 mGy/h) with accumulative low (≤100 mGy) or high (>100 mGy) dose radiation exposure. Our main goal of this study was to examine if different low dose rates of chronic pre- and/or post-natal radiation exposure with accumulative high doses could induce hippocampal cellular, mRNA, and miRNA changes leading to neuropsychiatric disorders. The comprehensive mouse phenotypic traits, organ weight, pathological, and blood mRNA and miRNA changes were also studied. Using different approaches including SmithKline, Harwell, Imperial College, Royal Hospital, Phenotype Assessment (SHIRPA), neurobehavioral tests, pathological examination, immunohistochemistry, mRNA and miRNA sequencing, and real-time quantitative polymerase chain reaction (qRT-PCR) validation, we found that in prenatally irradiated (100 mGy/d for 18 days with an accumulative dose of 1.8 Gy) 1-year-old mice, no cellular changes, including immature neurons in the subgranular zone, mature neurons and glial cells in the hilus of the dentate gyrus and development of cognitive impairment, neuropsychiatric disorders, occurred. However, a significant reduction in body weight and mass index (BMI) was indicated by the SHIRPA test. A reduced exploratory behavior was shown by an open field test. Organ weights showed significant reductions in the testes, kidneys, heart, liver and epididymides with no abnormal pathology. mRNA and miRNA sequencing and qRT-PCR validation revealed the upregulation of Rubcnl and Abhd14b, and downregulation of Hspa1b, P4ha1, and Banp genes in both the hippocampus and blood of mice prenatally irradiated with 100 mGy/d. Meanwhile, downregulation of miR-448-3p and miR1298-5p in the hippocampus, miR-320-3p, miR-423-5p, miR-486b-5p, miR-486b-3p, miR-423-3p, miR-652-3p, miR-324-3p, miR-181b-5p, miR-let-7b, and miR-6904-5p in the blood was induced. The target scan revealed that Rubcnl is one of the miR-181b-5p targets in the blood. We, therefore, concluded that prenatal chronic irradiation with a low dose rate of 100 mGy/d and accumulative dose of 1.8 Gy or below might not induce significant adverse health effects on the offspring. Further study of different low dose rate radiation exposures with accumulative high doses may provide threshold doses for authorities or regulators to set new radiation safety guidelines to replace those extrapolated from acute high dose/dose rate irradiation to reduce unnecessary emergency evacuation or spending once a nuclear accident or leakage occurs.

## 1. Introduction

Acute high dose/dose-rate dependent prenatal irradiation-induced adverse health effects, such as neural tube defects, microcephaly, severe intellectual disability, cancers, and unprovoked seizures have been seen due to radiotherapy [1,2,3,4] and atomic bomb exposure in Hiroshima and Nagasaki [5,6,7]. Acute exposure to low doses at a high dose rate, as seen in prenatal diagnostic X-ray exposures, may not significantly increase the risk of cancer development [8] except for leukemia, as reported by Schulze-Rath [9] and Wakeford [10]. While Down’s syndrome [11], trisomy 21 [12,13,14], neural tube defects, and microcephaly have been reported after Chernobyl chronic radiation exposure in humans [15,16,17], and microcephaly in the birds exposed to Chernobyl chronic radiation [18] and in monkeys exposed to the Fukushima nuclear accident [19], no individual dose/dose rate has been reported for mothers or embryos at different developmental stages of the prenatal period. These reports are not in agreement with the International Commission on Radiological Protection (ICRP)’s conclusion that irradiation throughout pregnancy with exposure rates of less than 0.05 Gy/day (or 2.08 mGy/h) are not measurably deleterious to the surviving offspring (ICRP report 90, Item 424). So far, limited lifetime or long-term chronic low dose rate ionizing radiation exposures in animal models have been performed, and inconsistent radiation effects including life span, carcinogenesis, and neoplastic death have been reported [20,21,22,23,24,25,26,27]. Our previous review shows the variations in the effects of acute prenatal radiation exposures on the progeny [4], and a recent literature review concludes that both epidemiological and experimental studies do not provide convincing evidence to support radiation-induced developmental defects in the progeny after exposure in utero [28]. Furthermore, there are no epidemiological or experimental studies on cellular changes in the brain after prenatal chronic low (<100 mGy) or high dose with low dose rate (<6 mGy/h) radiation exposure. In the present study, neurological, neurocognitive, and neuropsychiatric changes were examined in prenatal chronic continuous low dose rate irradiated progeny with SmithKline, Harwell, Imperial College, Royal Hospital, Phenotype Assessment (SHIRPA), novel object recognition, open field, forced swim, and tail suspension tests, respectively. Using different brain cell markers, including newly generated immature neuronal marker doublecortin (DCX), mature neuronal marker neuronal nuclear protein (NeuN), astrocyte marker glial fibrillary acidic protein (GFAP), microglial marker ionized calcium-binding adapter molecule 1 (IBA1), and oligodendrocyte precursor cell marker platelet-derived growth factor receptor alpha (PDGFRα), we aimed to investigate prenatal low dose rate radiation effect on the progeny’s cellular changes in the dentate gyrus of the hippocampus. We focused on the dentate gyrus of the hippocampus, as previous studies have shown obvious pathophysiological changes including neuronal loss, impairment of neurogenesis, and glial activation after different patterns of radiation exposure [4,28]. By immunohistochemical staining of DNA damage and repair marker H2A histone family member X (γH2AX), we examined if prenatal irradiation induced persistent DNA damage foci in the progeny’s brain. miRNA and mRNA sequencing and real-time quantitative reverse transcription PCR (qRT-PCR) analysis were performed to reveal irradiation-induced gene changes. Furthermore, a pathological study was also carried out for different body organs. The results of this study may provide evidence for a better understanding of the effect of nuclear accidents on the offspring of pregnant women staying near the accident site or exposed to nuclear fallout.

## 2. Materials and Methods

### 2.1. Animals and Irradiation

Six-week-old mice (SPF C57BL/6JJcl females and C3H/HeNJcl males) purchased from CLEA Japan Inc. (Tokyo, Japan) were used as parent stocks and bred as described by Gulay et al. [27]. Pregnant dams were continuously exposed to whole body irradiation using ^137^Cs gamma rays for 22 h/day(d), at daily doses of 1 or 20 mGy/d, from gestation day (GD) 0 (when vaginal plugs are detected) up to post-natal day 30 (approximately 48 consecutive days), to total accumulated doses of 48 and 960 mGy, respectively. A third irradiation group was exposed to 100 mGy/d from GD 0–18 to a total dose of 1.8 Gy. Age-matched non-irradiated pregnant dams were also included.

The absorbed doses by the pregnant dam are based on measurements made using thermoluminescence dosimeters (TLDs) inserted into the abdomen of mice as described by Shiragai et al. [29]. Pups were carefully counted (as soon as possible after birth) and were allowed to stay with their dams until weaning at 21 days (3 weeks) of age, at which time they were individually identified with ear notches, weighed, separated by sex, and group-caged (5 mice/cage).

A total of 64 male mice (n = 16/group) including age-matched non-irradiated controls used in the study were housed at the Low-Dose Radiation Effects Research Facility (LERF) of the Institute for Environmental Sciences (IES), Rokkasho, Aomori, Japan.

The entire study was conducted under similar SPF environmental conditions and husbandry practices; these include a 12 h light–dark cycle, weekly cage change, ad libitum feed and water supply, daily health monitoring or clinical inspection, and monthly monitoring of SPF status, as described previously [24]. All experiments were conducted according to legal regulations in Japan and following the Guidelines for Animal Experiments of the Institute for Environmental Sciences.

### 2.2. Behavioral Studies

A total of 32 B6C3F1 males (n = 16 non-irradiated and n = 16 exposed in utero to 100 mGy/d for 18 days), were tested using the SmithKline, Harwell, Imperial College, Royal Hospital, Phenotype Assessment (SHIRPA), Open Field (Locomotor), Novel Object Recognition, Forced Swim and Tail Suspension Tests.

#### 2.2.1. The SmithKline, Harwell, Imperial College, Royal Hospital, Phenotype Assessment (SHIRPA) Test

SHIRPA is a rapid battery of tests of motor activity, coordination, postural control, muscle tone, autonomic functions, and emotional reactivity, as well as reflexes dependent on visual, auditory, and tactile modalities. Individual scores in SHIRPA are sensitive in detecting phenotypes of several experimental models of neural disease, especially cerebellar degeneration and Alzheimer’s disease, and combined sub-scores have been useful in estimating the impact of vascular anomalies and exposure to infectious agents [30]. The RIKEN Modified SHIRPA (Version 4*) was used in the present study. It included 68 test items related to the evaluation of morphology, behavior, sensory response, and athletic ability. The test was executed as a series of six categories comprising “In the viewing jar”, to allow the mouse to get used to the environment and observe it; “In the Arena”, to observe the behavior in the arena; “Above the Arena 1”, to observe the behavior above the grid put on the arena; “On the Arena”, to observe the morphology and behavior on the grid put on the arena; “Above the Arena 2”, to observe the morphology and locomotion with the arena; and “Additional Comments”, to evaluate the behavior and somatotype. The test took approximately 15 min to complete for each mouse (Please refer to https://ja.brc.riken.jp/lab/jmc/shirpa/ (accessed on 21 July 2024) for the detailed protocol) [31].

#### 2.2.2. Open Field (Locomotor) Test

The open field test was performed in an empty and opaque box with the dimensions 50 cm × 50 cm. The arena is divided into 3 areas; center, corners, and outer, in the software to track the distance travelled and time spent in each area. The mouse was placed in the center area at the start of the test and was allowed to explore for 30 min, to observe their behavior. The ANY-maze software version 7.10 (ANY-maze, Wood Dale, IL, USA) detects the center of the mouse’s body, thus only detecting entry, and tracking time and distance once half of the mouse’s body is in the area.

#### 2.2.3. Novel Object Recognition Test

This test was carried out over 4 days: 2 habituation days, a training day, and a test day. Mice were placed in an empty box with the dimensions 50 cm × 25 cm for 10 min. Habituation and training were performed with 2 plastic macarons and 2 rubber square waffles placed on opposite sides of the box respectively. The test was conducted 24 h after the training day, where one of the square waffles from training was replaced with a novel, circular plastic bowl object. Between each animal, both the arena and toys were wiped with clidox first, and then ethanol. Using the ANY-maze investigation zone feature (ANY-maze, Wood Dale, IL, USA), we set a zone around the object with a radius equal to the mouse’s body length (2–3 cm). The software will detect and measure the time the mouse spent in the 2 different object zones.

#### 2.2.4. Forced Swim Test

A cylindrical tank of 20 cm diameter was used for the forced swim test with a water temperature of 24–26 °C. Mice were placed in water and movement was recorded for 8 min. Immobility was detected by the ANY-maze (ANY-maze, Wood Dale, IL, USA) when there were no limb movements for 3 s or more. Immobilization is regarded as an indication of depression-like behavior [32].

#### 2.2.5. Tail Suspension Test

This test requires the mice to be suspended by their tails, which were taped to a hook. Their movements were recorded with ANY-maze (ANY-maze, Wood Dale, IL, USA) for 6 min with immobility detected when there were no limb movements for 3 s or more. The time spent as immobile was recorded for consideration for mice’s depressive-like behavior.

### 2.3. Pathological Examination

All 64 B6C3F1 male mice, 16 from each group of the control, and experimental groups irradiated with 1, 20, and 100 mGy/day, were sacrificed at approximately 1 year of age by carbon monoxide asphyxiation, after which blood samples were collected via cardiac puncture and then subjected to necropsy (gross examination). Whole blood, 0.5 mL, was transferred into 2 mL tubes pre-loaded with RNAlater solution and stored frozen at −80 °C until analyzed. Organs except the brain were collected, examined, weighed, and fixed in 10% neutral buffered formalin for histopathological examination based on a standard protocol [25]. When deemed necessary, additional tissue samples were collected from neoplasms and organs or tissues with gross abnormalities and special histochemical procedures were performed for diagnostic purposes. Histopathological examination was performed blindly, and neoplasms were classified based on the proposed nomenclatures of WHO/IARC [33] and the NTP [34] as described previously [25]. Multiple primary neoplasms and pathologies were treated as in the previous lifespan study [25], wherein multiple (including multiple or metastatic foci) neoplasms of the same type were counted only once. All neoplasms were counted into the overall incidence.

The whole brain was dissected and separated sagittally into the left and right hemispheres. The right hemisphere was fixed in 4% paraformaldehyde for 24 h, then transferred to 30% sucrose in 0.1 M phosphate buffer (pH 7.4) for immunohistochemistry. The hippocampus was dissected from the left hemisphere and stored frozen at −80 °C until it was processed for RNA extraction.

### 2.4. Immunohistochemical Staining of the Hippocampus

Seven to nine serial sagittal sections of the left hemisphere of the brain (40 μm thick) from 8 non-irradiated mice, 7 mice from the 100 mGy/d group, and 5 mice each from the 1 and 20 mGy/d groups were placed in 24-well plates with PBS, and immunostained for DCX, NeuN, PDGFRα, GFAP, IBA1, and γH2AX according to our previous study [35]. After treatment with 3% H_2_O_2_ (Sigma-Aldrich Pte Ltd., Singapore) and blocking with serum (Vector Laboratories Inc., Burlingame, CA, USA), free-floating sections were incubated with goat primary antibody against DCX (1:500; Santa Cruz Biotechnology Inc., Santa Cruz, CA, USA, Catalog No: SC-8066), rabbit primary antibody against NeuN (1:1000; Invitrogen, MA, USA, Catalog No: PA5-37407), and PDGFRα (1:200) (Catalog No: 3174S), GFAP (1:200) (Catalog No: 12389S), IBA1 (1:200) (Catalog No: 17189S), and γH2AX (1:200) (Catalog No: 9718S) (Cell Signaling Technology, Danvers, MA, USA) overnight. The sections were then washed and incubated with respective donkey anti-goat (Catalog No: ab6884) (Abcam Inc., Waltham, MA, USA) and goat anti-rabbit (Catalog No: 14789S) (Cell Signaling Technology, Danvers, MA, USA) secondary antibodies (1:200) followed by avidin-biotin complex (ABC) reagent (Vector Laboratories Inc., Burlingame, CA, USA). After reaction in 3,3′-diaminobenzidine (DAB) peroxidase substrate (Vector Laboratories Inc., Burlingame, CA, USA), the sections were then washed, mounted, counterstained, and covered.

The immunostained sections were examined and photographed (Leica Microsystems GmbH, Wetzlar, Germany) and the Stereology System (Stereology Resource Center, Biosciences Inc., Tampa, FL, USA) was used to unbiasedly analyze the number of NeuN, PDGFRα, and GFAP immunopositive cells in the hilus, as well as IBA1 immunopositive cells in the hilus and stratum granulosum, indicated as the number/volume (mm^3^). DCX immunopositive cells in the subgranular zone were counted and indicated as a number per subgranular length (mm).

### 2.5. RNA Extraction from the Hippocampus and Whole Blood

RNA extraction from the hippocampus was performed in 6 non-irradiated control and 6 prenatally irradiated mice from the 100 mGy/d group using the miRNeasy Mini Kit (Qiagen, Hilden, Germany). The hippocampus was homogenized in 700 µL QIAzol lysis reagent, placed at room temperature for 5 min, added with 140 µL chloroform, and shaken vigorously for 15 s. The tube was centrifuged for 15 min at 12,000× *g* at 4 °C. After centrifugation, the samples were separated into 3 phases. The upper colorless aqueous phase containing RNA was collected into a new tube, and then mixed with 1.5 volumes of 100% ethanol. The above mixture was loaded into an RNeasy Mini spin column, and centrifuged at ≥8000× *g* for 15 s. The column was washed and centrifuged. RNA from the column membrane was finally eluded with 40 µL RNase-free water.

RNA from whole blood was isolated using Mouse RiboPure™-Blood RNA Isolation Kit (Life Technologies Holdings Pte Ltd., Singapore). Mouse blood was collected in a tube with pre-loaded RNAlater solution, and centrifuged for 3 min at 15,000× *g*. The supernatant was removed. The cell pellet was reconstituted by adding a lysis solution, and vortexed vigorously, followed by 200 µL 3 M sodium acetate and 1.5 mL acid phenol, with chloroform added. The tube was centrifuged for 10 min at 2000× *g*. The aqueous upper phase was recovered and mixed with 1.2 volumes of 100% ethanol. The sample was then vacuum-filtered through a filter cartridge, washed, and eluted with 150 µL nuclease-free water. RNA concentration and integrity were checked using the Nanodrop and Bioanalyzer system (Agilent Technologies, Santa Clara, CA, USA) before being subjected to miRNA sequencing and qPCR analysis.

### 2.6. Systematic miR Sequencing (miRSeq) and mRNA Sequencing Analysis

miRSeq and mRNA sequencing of the hippocampus and blood from 3 of 6 extracted samples from mice irradiated with 100 mGy/day were carried out using the DNB SEQ platform (BGI, Beijing, P.R. China); detected 1976 miRs, and 19,039 mRNAs were further analyzed by DESeq2 method.

### 2.7. Real-Time Quantitative Reverse Transcription PCR (qRT-PCR) Analysis of miR

RNA was first reversely transcribed using the miScript II RT kit (Qiagen, Hilden, Germany). The 20 µL reaction mixture, including 4 µL 5× HiSpec buffer, 2 µL 10× nucleotide mix, 2 µL reverse transcripts mix, 5 µL template RNA, and 7 µL nuclease-free water, was incubated at 37 °C for 1 h followed by 95 °C for 5 min. The resulting cDNA was then diluted by adding 80 µL of nuclease-free water and stored at −80 °C until analysis.

20 µL of master mix, for real-time PCR, was prepared as follows: 2 µL diluted cDNA, 10 µL 2× miScript SYBR green PCR master mix, 2 µL 10× miScript universal primer and 2 µL primer for target miR (Table 1), and 4 µL nuclease-free water. Samples were denatured at 95 °C for 15 min, followed by 40 cycles of denaturation at 94 °C for 15 s, annealing at 55 °C for 30 s, and extension at 70 °C for 30 s. PCR amplification was carried out in QuantStudio 6 Real-Time PCR Systems (Thermo Fisher Scientific, Waltham, MA, USA). Fluorescence data were then collected. The expression of miR-68 was used as an internal control.

### 2.8. Real-Time RT-PCR Analysis for mRNA

For real-time RT-PCR analysis of mRNA, RNA was reverse transcribed using Maxima first-strand cDNA synthesis kits (Thermo Fisher Scientific, Waltham, MA, USA). A total of 1 µg RNA was added to 4 µL 5× Reaction Mix, 2 µL Maxima Enzyme Mix, and topped up to 20 µL with nuclease-free water. The tubes were thereafter incubated at 25 °C for 10 min, followed by 50 °C for 45 min and 85 °C for 5 min. The resulting cDNA was then diluted by adding 100 µL of nuclease-free water and stored at −20 °C.

A master mix of 20 µL for real-time PCR was prepared as follows: 2 µL diluted cDNA, 10 µL 2× Maxima SYBR Green qPCR Master Mix, 2 µL 10× forward and reverse primers for target genes (Table 2), and 4 µL nuclease-free water.

PCR amplification was carried out in QuantStudio 6 Real-Time PCR Systems (Thermo Fisher Scientific, Waltham, MA, USA). The samples were initially denatured at 95 °C for 10 min, followed by 40 cycles of the following: denaturation at 95 °C for 15 s, annealing at 60 °C for 30 s, and extension at 72 °C for 30 s. The fluorescence data were collected after the extension step. The expression of GAPDH was used as internal control.

### 2.9. Statistical Analyses

Student’s *t*-test was used to compare behavioral changes between the non-irradiated control and irradiated mice. Fischer’s exact tests were used to analyze the non-neoplastic lesions and neoplasm incidence. Levels of significance for incidence rates of non-neoplastic lesions and neoplasms were chosen as *p* < 0.05. For detected miRs and mRNAs analysis, the parameters for calculating the significantly differential expression of DEseq2 are |log2FC| > 0.585 and *p* < 0.05. To compare the immunostained DCX, NeuN, PDGFRα, GFAP, and IBA1 cells among the control, pre-, and/or post-natally irradiated mice, One-Way ANOVA followed by a Post Hoc test was performed. Brown–Forsythe test and Bartlett’s test were used to check data normality and homogeneity respectively. Turkey test was performed as a post hoc test to compare the mean of each group of mice with the mean of every other group.

## 3. Results

### 3.1. Behavioral Studies

#### 3.1.1. SHIRPA Test

**Figure 1:**
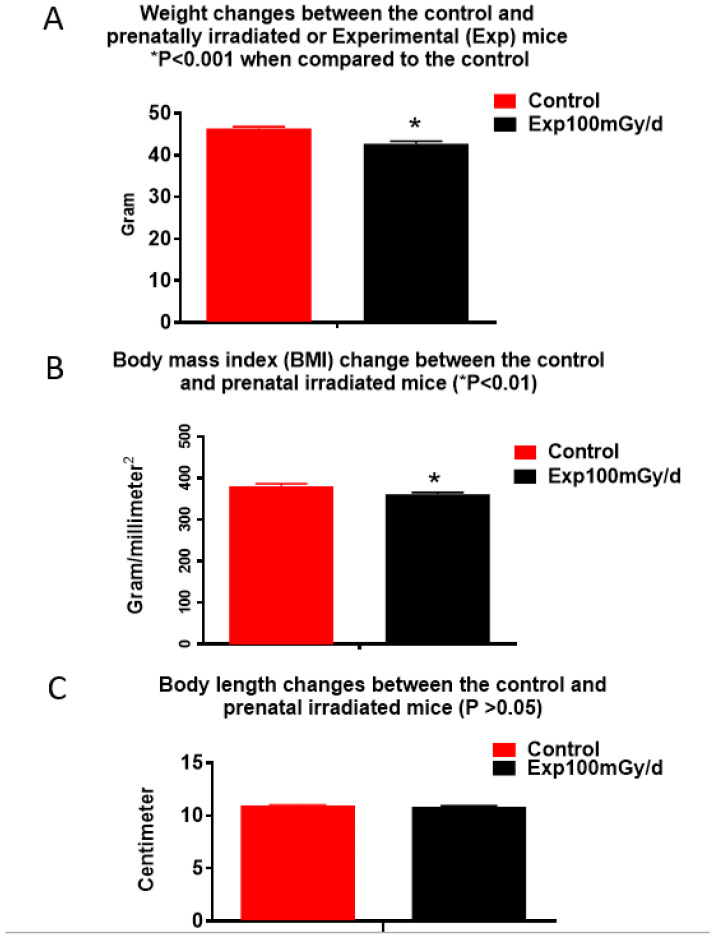
Prenatal irradiation reduces body weight and BMI in mice without affecting body length. (**A**) The SHIRPA test indicates a significant reduction in body weight (n = 16, * *p* < 0.001 when compared to the control). (**B**) Body mass index (BMI) is reduced significantly (n = 16, * *p* < 0.01 when compared to the control) in mice prenatally irradiated with a dose rate of 100 mGy/d for the entire gestation period. (**C**) No difference in body length is observed (n = 16, *p* > 0.05 when compared to the control).

The results of the SHIRPA test (68 parameters) in male B6C3F1 at 1 year after prenatal irradiation showed significant differences in body weight and body mass index (BMI) between irradiated mice (100 mGy/d) and the non-irradiated controls (Figure 1). Here, BMI is defined as

$$BMI=\frac{W}{L^{2}}\times 1000$$

where *W* is body weight in grams and *L* is body length in mm. There was no significant change in other parameters examined (for details, please refer to the website https://ja.brc.riken.jp/lab/jmc/mouse_clinic/SOPs/Classification_by_Platform/Other/RIKENMPP_001_004_02_modified_SHIRPA_v4.xml, accessed on 21 July 2024). The results suggest that prenatal irradiation with 100 mGy/d for 18 days does not induce any impairment of sensorimotor function nor emotional reactivity 1 year after radiation exposure.

#### 3.1.2. Open Field Test

**Figure 2:**
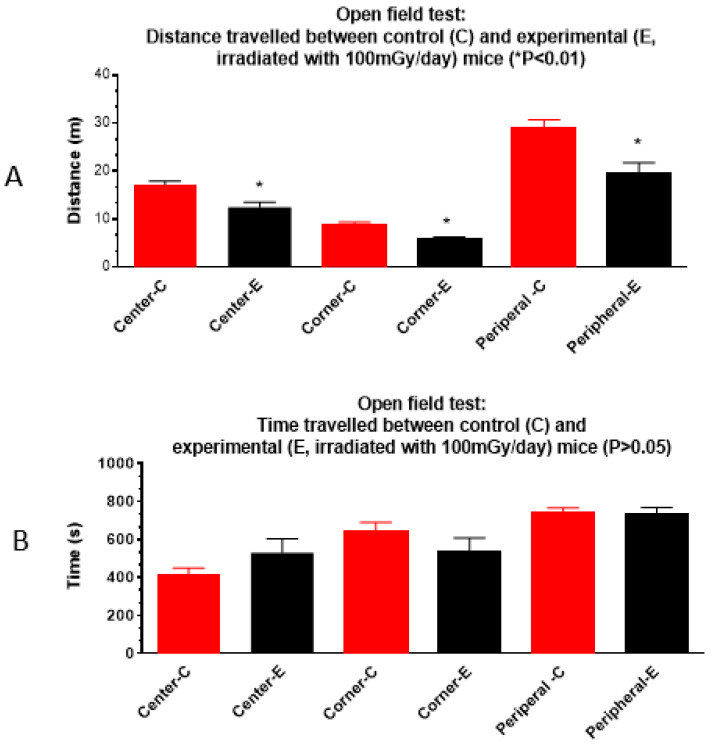
Prenatal irradiation with 100 mG/day affects mouse locomotor abilities in the Open field test: The distance irradiated animals travelled in each center, peripheral, and corner area (black bar) is reduced (**A**) (* *p* < 0.01 when compared to the control), but the time spent is similar (**B**) between the control and the prenatally irradiated mice (n = 16, *p* > 0.05). Center-C: the distance control animals travelled in the center area, Center-E: the distance experimental or irradiated (100 mGy/d) animals travelled in the center area, Corner-C: the distance control animals travelled in the corner area, Corner-E: the distance irradiated animals travelled in the corner area, Peripheral-C: the distance control animals travelled in the peripheral area, Peripheral-E: the distance irradiated animals travelled in the peripheral area (* *p* < 0.01).

The open field test showed that the average distance the irradiated mice travelled to different areas of the box was reduced significantly (*p* < 0.05) (Figure 2A) compared to the non-irradiated controls, although the time spent in each area was not significantly different (Figure 2B). Results suggest that irradiated mice had decreased locomotor activity or reduced exploratory behavior compared to the non-irradiated control mice.

#### 3.1.3. Novel Object Recognition, Forced Swim, and Tail Suspension Tests

**Figure 3:**
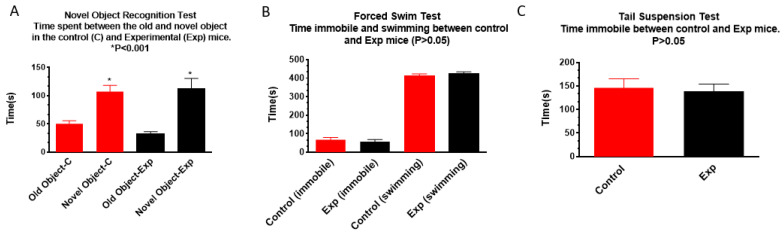
Prenatal irradiation does not affect animal novel object recognition (**A**), nor induce longer immobile time in the forced swim (**B**) and tail suspension (**C**) tests: in (**A**), both control and prenatally irradiated mice spend more time with a novel object (n = 16, * *p* < 0.05 when compared to the control). In (**B**,**C**), there is no difference in immobile time, and in (**B**), no difference in swimming time is observed between the control and prenatally irradiated mice (n = 16, *p* > 0.05 when compared to the control).

Results of the novel object recognition test showed that both the non-irradiated control and the irradiated groups spent the same amount of time with the novel object, suggesting that prenatal irradiation did not impair novel object recognition (Figure 3A). Forced swim (Figure 3B) and tail suspension (Figure 3C) tests did not show significant differences between groups in immobile time, suggesting that these irradiated mice did not develop depression.

### 3.2. Pathology

**Figure 4:**
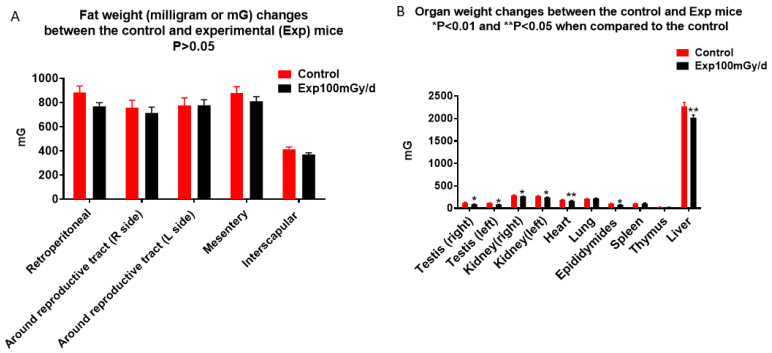
Prenatal irradiation does not affect animal body fat deposits (**A**) but reduces organ weight (**B**): Irradiated mice have similar fat deposits in different parts of the body (**A**) (*p* > 0.05), but reduced organ weight in the testis, kidney, epididymides (* *p* < 0.01), heart, and liver (n = 16, ** *p* < 0.05 when compared to the control (**B**)).

As the SHIRPA test indicated a significant reduction of body weight in mice prenatally irradiated with 100 mGy/d, adipose tissue deposits and different organs including left and right testis, kidney, heart, liver, lung, epididymides, spleen, thymus were collected, and weighed, the results did not show a significant reduction in the weight of adipose tissue deposits between the irradiated and control mice (Figure 4A). However, there was a significant reduction of the weight of testis (control, right 130 ± 7.11, Exp right 92 ± 2.08; control left 119 ± 2.07), kidney (control right 293.63 ± 6.89, Exp right 266.19 ± 3.71), heart (control 192.44, Exp 166.14 ± 10.35), liver (control 2269.44 ± 92.63, Exp 2019.50 ± 60.02) and epididymides (control 105.63 ± 6.55, Exp 80 ± 1.28) (Figure 4B).

Histopathological examination showed that the most common neoplastic lesions in males were hepatocellular adenoma, hepatocellular carcinoma, and lung adenoma. However, there was no significant difference in the incidence of these lesions between the non-irradiated and irradiated groups (Table 3). Non-neoplastic lesions observed included subcapsular cell hyperplasia of the adrenal gland, valvular endocardiosis in the heart, cytoplasmic vacuolation and granular degeneration in the liver, and inflammation in the lung, but incidence rates were not significantly different between the non-irradiated and irradiated groups. The study indicates that continuous low dose rate irradiation during the gestation period (gestation days 0–18) and the juvenile period (1–30 days) does not lead to the development of neoplasms or non-neoplastic lesions at the examined endpoint.

### 3.3. Immunohistochemistry

**Figure 5:**
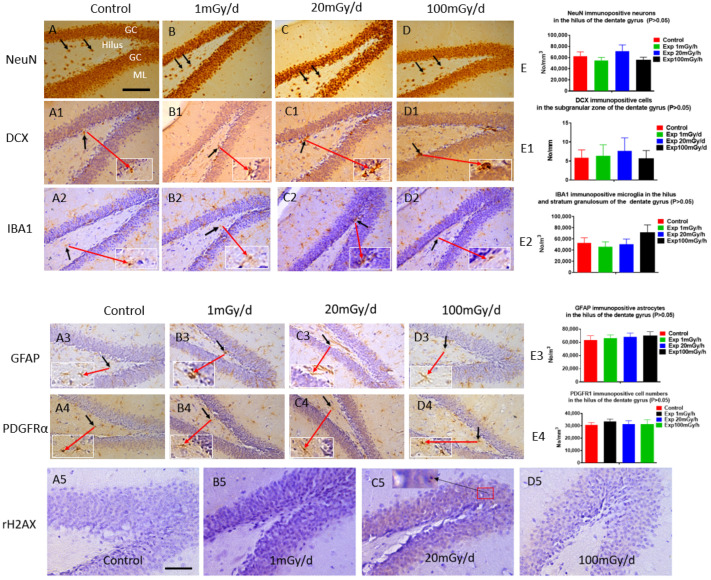
Prenatal irradiation does not induce any cellular changes in the dentate gyrus, nor result in persistent DNA damage demonstrated by the immunohistochemical staining: There is no obvious change in the number of NeuN immunopositive mature neurons (indicated by a black arrow; an enlarged cell is indicated by a red arrow in the insert) in the hilus (**A**–**E**), DCX immunopositive immature neurons (indicated by a black arrow; an enlarged cell is indicated by a red arrow in the insert) (**A1**–**E1**) in the subgranular zone, IBA1 immunopositive microglia (indicated by a black arrow; an enlarged cell is indicated by a red arrow in the insert) in the hilus and the granule cell layer (GC) (**A2**–**E2**), GFAP immunopositive astrocytes (indicated by a black arrow; an enlarged cell is indicated by a red arrow in the insert) (**A3**–**E3**), and PDGFRα immunopositive oligodendrocyte precursor cells (indicated by a black arrow; an enlarged cell is indicated by a red arrow in the insert) (**A4**–**E4**) among the control and experiment mice irradiated with 1, 20, and 100 mGy/d, respectively (*p* > 0.05). γ-H2AX immunostaining shows DNA damage foci occasionally in the granule cells (insert in (**C5**)). GC: granule cell layer, ML: molecular layer. Scale bar = 100 µm in (**A**): applies to (**A1**–**A4**,**B**–**B4**,**C**–**C4**,**D**–**D4**), Scale bar = 50 µm in (**A5**): applies to (**B5**–**D5**).

Immunohistochemical study did not show significant differences in the number of mature neurons (NeuN, Figure 5A–E) in the hilus, immature neurons (DCX, Figure 5(A1)–(E1)) in the subgranular zone, microglia (IBA1, Figure 5(A2)–(E2)) in both hilus and the stratum granulosum, astrocytes (GFAP, Figure 5(A3)–(E3)) or oligodendrocyte precursor cells (PDGFRα, Figure 5(A4)–(E4)) in the hilus of the dentate gyrus of the hippocampus between the non-irradiated control and irradiated groups.

γ-H2AX immunostaining did not detect persistent DNA damage foci 1 year after in pre-and/or post-natally irradiated male mice (Figure 5(A5)–(D5)).

### 3.4. Systematic miR Sequencing (miRSeq) and mRNA Sequencing Analysis

**Figure 6:**
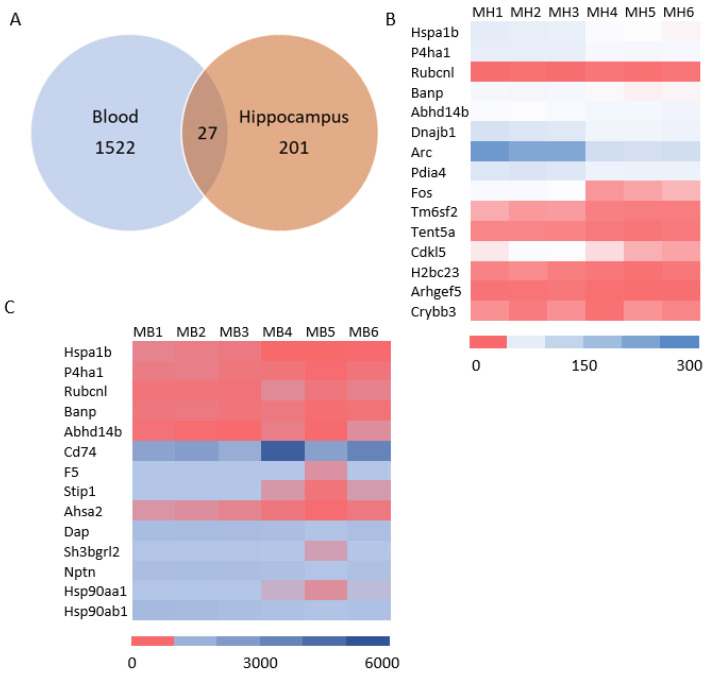
Prenatal irradiation induces changes of mRNA in either both the hippocampus and blood or in the hippocampus or blood: (**A**) Venn diagram of differentially expressed mRNA between hippocampus and blood; (**B**,**C**) heatmap of selected mRNAs which are differentially expressed in the hippocampus and blood between the control and irradiated mice respectively (n = 3, |log2FC| > 0.585 and *p* < 0.05). MH1–3: three control male hippocampus (MH) samples; MB4–6: three irradiated experimental male hippocampus samples; MB1–3: three control male blood (MB) samples; MB4–6, irradiated experimental male blood samples.

**Figure 7:**
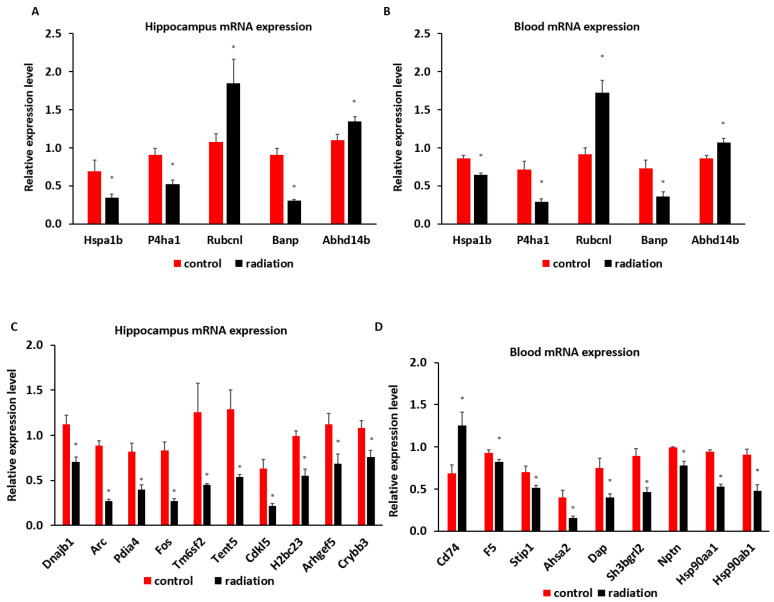
qRT-PCR indicates a down-regulation of *Hspa1b*, *P4ha1*, *Banp*, and an up-regulation of *Rubanl* and *Abhd14b* in both the hippocampus (**A**) and whole blood (**B**) (n = 6, * *p* < 0.05 when compared to the control). In the hippocampus, there is a significant down-regulation of *Dnajb1*, *Arc*, *Pdia4*, *Fos*, *Tm6sf2*, *Tent5*, *Cdkl5*, *H2bc23*, *Arhgef5*, *Crybb3* (**C**), whereas *CD74* is up-regulated, *F5*, *Stip1*, *Ahsa2*, *Dap*, *Sh3bgrl2*, *Nptn*, *Hsp90aa1, and Hsp90ab1* genes are down-regulated in whole blood (**D**). (n = 6, * *p* < 0.05 when compared to the control).

**Figure 8:**
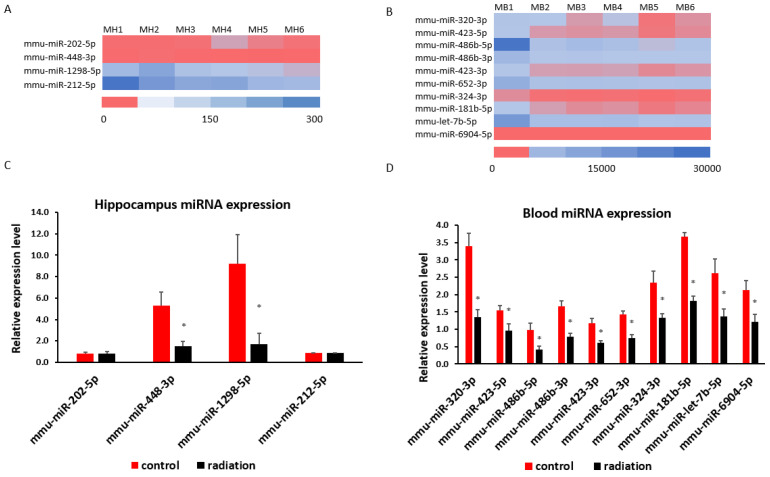
Prenatal irradiation induces miRNA changes in the hippocampus or blood: heatmap of miRNA sequencing results in the hippocampus is indicated in (**A**), and blood in (**B**) (n = 3, |log2FC| > 0.585 and *p* < 0.05); qRT-PCR indicates a down-regulation of miR-448-3p and miR-1298-5p in the hippocampus (**C**), and miR-320-3p, miR-423-5p, miR-486b-5p, miR-486b-3p, miR-423-3p, miR-652-3p, miR-324-3p, miR-181b-5p, miR-let-7b, and miR-6904-5p in whole blood (**D**). (n = 6, * *p* < 0.05). MH1–3: three control male hippocampus (MH) samples; MB4–6: three irradiated experimental male hippocampus samples; MB1–3: three control male blood (MB) samples; MB4–6, irradiated experimental male blood samples.

mRNA sequencing analysis showed 1549 differentially expressed mRNAs in whole blood and 228 differentially expressed mRNAs in the hippocampus. The Venn analysis indicated that 27 mRNAs were altered in both whole blood and hippocampus (Figure 6A). Of the differentially expressed mRNA, 20 or 25 highly and significantly expressed mRNA with the same up- or down-expression trend from the hippocampus or whole blood, respectively, were validated with qRT-PCR. Some differentially expressed mRNAs from mRNA sequencing results were summarized by heatmap in the hippocampus and blood (Figure 6B,C). The PCR and mRNA sequencing results showed that RUN and cysteine-rich domains containing beclin 1 interacting proteins like (*Rubcnl*) and alpha/beta hydrolase domain-containing protein 14B (*Abhd14b*) genes were up-regulated, whereas heat shock protein family A member 1B (*Hspa1b*), prolyl 4-hydroxylase subunit alpha 1 (*P4ha1*), and BTG3 associated nuclear protein (*Banp*) genes were down-regulated in both the hippocampus (Figure 6B and Figure 7A) and whole blood (Figure 6C and Figure 7B). In the hippocampus, significant down-regulation of *Dnajb1*, *Arc*, *Pdia4*, *Fos*, *Tm6sf2*, *Tent5*, *Cdkl5*, *H2bc23*, *Arhgef5*, *Crybb3* (Figure 6B and Figure 7C) were observed. In whole blood, *CD74* was up-regulated while *F5*, *Stip1*, *Ahsa2*, *Dap*, *Sh3bgrl2*, *Nptn*, *Hsp90aa1and Hsp90ab1genes* were down-regulated (Figure 6C and Figure 7D). There was no change in the expressions of *F5*, *Dusp6*, *Egr2*, *Hspb1*, or *CD74* in the hippocampus (Figure S1A), nor of *Vmn1r58*, *Scd1*, *Cd59b*, *Slc5a3*, *Ccdc117*, *Cish*, *Fosb*, *Gem*, *H2bc24*, *Fam122a*, or *Sesn3* in whole blood (Figure S1B) demonstrated. Of 4 differentially expressed miRNAs (miR-202-5p, miR-448-3p, miR1298-5p, and miR-212-5p) in the hippocampus from sequencing analysis (Figure 8A), qRT-PCR validated the down-regulation of miR-448-3p and miR1298-5p (Figure 8B). Of the 75 differentially expressed miRNAs in the whole blood from sequencing analysis, 16 highly or significantly expressed miRNAs were tested by qRT-PCR. miR-320-3p, miR-423-5p, miR-486b-5p, miR-486b-3p, miR-423-3p, miR-652-3p, miR-324-3p, miR-181b-5p, miR-let-7b, and miR-6904-5p were down-regulated both in miRNA sequencing and qRT-PCR (Figure 8C,D). However, miR-122-5p, miR-744-5p, miR-let-7d-3p, miR-328-3p, miR-151-3p, and miR-296-5p were not changed (Figure S1C). The target scan indicated that among whole blood miRNA and mRNA changes, *Rubcnl* is one of the miR-181b-5p targets. 

## 4. Discussion

### 4.1. Main Findings

Prenatal chronic low dose rate irradiation with 100 mGy/d significantly reduced body weights and BMI, but no other change was observed when the SHIRPA test was performed on the offspring male B6C3F1 mice at the age of 1 year old. These mice also showed a reduction in exploratory behavior, but no memory loss and depressive-like behavior as tested by the novel object recognition, forced swim, and tail suspension tests, respectively. Histopathological examinations showed neoplastic and non-neoplastic lesions in various organs such as the liver, lung, heart, adrenal glands, and kidney; there was no significant difference among the non-irradiated control and irradiated groups exposed to 1, 20, and 100 mGy/d, although some organ weight was reduced. The chronic low dose rate irradiation did not induce any significant cellular changes in the dentate gyrus, including newly generated neurons in the sub-granular zone, mature polymorphic neurons, microglia, astrocytes, and oligodendrocyte precursor cells in the hilus. Prenatal irradiation with 100 mGy/d (accumulated dose of 1.8 Gy) also did not induce persistent DNA damage as shown by γ H2AX immunostaining in the granule cells of the dentate gyrus, suggesting that prenatal irradiation-induced DNA damage may be repaired by the age of 1 year old. mRNA and miRNA sequencing and qRT-PCR validation revealed the changes of some mRNAs and miRNAs; in particular, up-regulation of *Rubcnl* and *Abhd14b*, and down-regulation of *Hspa1b*, *P4ha1*, and *Banp* genes in both the hippocampus and whole blood of mice irradiated with 100 mGy/d. In the whole blood, Rubcnl may be one of the miR-181b-5p targets as indicated by the target scan.

### 4.2. Pre- and Post-Natal (First 31 Days) Irradiation with a Low Dose Rate of 1 mGy/d and 20 mGy/d Did Not Induce Obvious Pathological Changes in the Brain and Other Organs

A previous report on in utero low dose-rate gamma-ray exposure of 20 mGy/d for the entire gestation period (accumulated dose: 360 mGy) in B6C3F1 mice did not cause any significant effect in pups when compared to the nonirradiated controls up to 10 weeks of age [27]. Further study indicated that in utero low dose-rate gamma ray exposures to 0.05, 1.0, and 20 mGy/d did not affect the reproductive parameters such as litter size and weaning rates among the three groups. Mean life spans and tumor spectra were not significantly different among the groups irradiated with 0.05, 1.0, and 20 mGy/d compared to the non-irradiated controls [26]. Results of these two studies suggest that chronic in utero exposure to gamma rays at dose rates below 20 mGy/d for the entire gestation period does not induce obvious harmful health effects, at both early and late adult life stages of B6C3F1 mice. The negative results or no adverse effect from combined pre- and post-natal chronic irradiation with 1 mGy/d (accumulated dose of 48 mGy) or 20 mGy/d (accumulated dose of 960 mGy) in 1-year-old (middle aged) mice further support the conclusion that prenatal irradiation with 20 mGy/d does not induce harmful health effects in early adulthood or at middle age until natural death. This conclusion was also consistent with previous studies showing that in utero r-ray irradiation for the entire gestation period (days 1 to 18) in mice [36,37] and rats [38], at dose rates ranging from 25 mGy/d to 124 mGy/d produced no harmful effect. However, the induced histopathological changes at different life stages of the animal after prenatal exposure to medium dose rates of 200 mGy/d and 400 mGy/d to total accumulated doses of 3600 mGy and 7200 mGy [26], respectively, suggest that a threshold dose/dose rate may exist from 20 to 200 mGy/d when one of the parameters is fixed.

### 4.3. Prenatal Irradiation with a Dose Rate of 100 mGy/d (4.55 mGy/h, Total Accumulated Dose = 1.8 Gy) Did Not Induce Obvious Pathophysiological Changes in Middle-Aged Mice

While the present study showed that prenatal chronic low dose rate irradiation with 100 mGy/d significantly reduced body weights, BMI, and some organ weights, the pathological study did not indicate any abnormal changes. Further study with a large sample size may confirm whether this dose rate induces animal body physical changes. No detectable changes in the offspring’s motor activity, coordination, postural control, muscle tone, autonomic functions, emotional reactivity, reflexes (dependent on visual, auditory, and tactile modalities) as well as neurobehavior, including cognition and neuropsychiatry after the prenatal chronic low dose-rate irradiation with 100 mGy/d, suggest that animals may have a normal life, at least during the first half of their lifespan. At the molecular level, the significance of the up-regulation of *Rubcnl* and *Abhd14b*, and down-regulation of *Hspa1b*, *P4ha1*, and *Banp* genes in both the hippocampus and whole blood, up- or down-regulation of other mRNA and miRNA in either the hippocampus or whole blood of mice prenatally irradiated with 100 mGy/d remain unknown due to limited functional study of the roles of these mRNAs and miRNAs.

*Rubcnl*, a recently identified novel accessory protein of PtdIns3K complexes, positively regulates autophagosome maturation and is primarily localized in the endoplasmic reticulum (ER) and autophagic structures. It antagonizes RUBCN to stimulate PIK3C3/Vps34 kinase activity and to recruit PtdIns3K and HOPS complexes to the autophagosome for their site-specific activation by anchoring to the autophagosomal SNARE STX17. Hepatocyte-specific *Rubcnl* ablation in mice results in impaired autophagy flux, glycogen and lipid accumulation, and liver fibrosis, whereas overexpression of *Rubcnl* in mouse livers alleviates non-alcoholic fatty liver disease [39]. Alpha/beta hydrolase domain-containing protein 14B (*Abhd14b*) belongs to the α/β hydrolase superfamily of enzymes, which are known for their diverse roles in lipid metabolism, signal transduction, and cellular homeostasis; it is found in various tissues throughout the body, including the brain, liver, and adipose tissue, suggesting its potential roles in lipid metabolism, neuronal function, and possibly other cellular processes since enzymes in the α/β hydrolase family often act as lipases, esterases, or thioesterases, cleaving ester bonds in various lipid molecules. From a speculative point of view, the upregulation of Rubcnl and Abhd14b in the blood may play a beneficial role in alleviating the radiation-induced decrease of different organ weights and potential abnormal lipid metabolism due to their anti-fibrosis properties and involvement in lipid metabolism. The upregulation of Rubcnl and Abhd14b in the hippocampus remains to be further investigated.

*The Hspa1b* gene and its protein HSP70-2 are involved in oxidative stress response, and an indel polymorphism in *Hspa1b* may be associated with the risk of sudden cardiac death [40]. Maternal separation (MS) enhanced the expression of *Hspa1b* mRNA in the blood and medial prefrontal cortex (mPFC) of juvenile and pre-adolescent rats [41] and is accompanied by an increase in the Hspa1a/1b protein levels in the mPFC and hippocampus of juvenile rats that persisted in the mPFC until adulthood. These changes suggest that *Hspa1b* may be a potential candidate peripheral and brain biomarkers of early-life stress (ELS)-induced changes in brain functioning [34], hepatocellular carcinoma [42], and in the early prediction and progression of Type 2 diabetes mellitus (T2DM) [43]. The level of *Hspa1b* in whole blood is also a sensitive marker for distinguishing tophi patients from healthy people [44]. Prolyl 4-hydroxylase subunit alpha 1 (*P4ha1*) has been identified as a valuable transcriptional genetic marker in female Sprague–Dawley rats’ blood, liver, and adrenal tissues under heat stress conditions [45]. *P4ha1* is one of the metabolic-related genes for predicting poor clinical prognosis and immune microenvironment in primary melanomas [46], lung adenocarcinoma [47], and squamous cell carcinoma of the head and neck [48]. *P4ha1* protects nasopharyngeal carcinoma (NPC) cells from erastin-induced ferroptosis by activating cytoplasmic 3-hydroxy-3-methylglutaryl-CoA synthase (HMGCS1), suggesting it may be a novel molecular marker of NPC ferroptosis resistance that results in poor prognosis, and that the P4ha1/HMGCS1 axis may be a new target for the treatment of NPC progression [49]. *P4ha1* may also be a potential biomarker in human atrial fibrillation [49] since hypoxia-induced endothelial *P4ha1* overexpression has been reported to enhance angiogenesis by promoting glycolytic metabolism reprogramming through the P4ha1/α-ketoglutarate (α-KG)/ten-eleven translocation 2 (Tet2)/fructose-1,6-biphosphatase (*Fbp1*) promoter pathway, leading to decreased *Fbp1*, and enhanced glycolytic metabolism, suggesting its therapeutic potential for post-ischemic tissue repair [50]. The *Banp* gene has been identified as a new prognostic biomarker of high-risk acute myeloid leukaemia (AML) [51]. Whether the down-regulation of *Hspa1b*, *P4ha1*, and *Banp* genes in whole blood and in the hippocampus in the present study is involved in the body’s self-protective mechanism in preventing low dose rate irradiation-induced early-life stress and cancers remains unknown. Given that limited studies have been carried out to investigate the functional changes of the miRNAs validated in this study, the significance of the down-regulation of miR-448-3p and miR1298-5p in the hippocampus and miR-320-3p, miR-423-5p, miR-486b-5p, miR-486b-3p, miR-423-3p, miR-652-3p, miR-324-3p, miR-181b-5p, miR-let-7b, and miR-6904-5p in whole blood needs to be further studied. Interestingly, among miRNA and mRNA changes in whole blood, Rubcnl is one of the miR-181b-5p targets by target scan. Whether the interaction of blood miR-181b-5p with Rubcnl is involved in animal weight changes induced by prenatal low dose rate radiation exposure remains to be further studied.

### 4.4. Low Dose/Dose Rate Radiation-Induced Animal Weight Changes

Low dose/dose rate radiation-induced animal weight changes varied from different studies. Postnatal irradiation of 3-month-old rats with combined r-rays (^137^Cs, 400 ± 20 mGy) with ^12^C (140 ± 14 mGy) induced significant weight loss [52]. However, chronic irradiation of C3H male mice (at the age of 10–11 weeks old) with a mixed field of neutrons and photons at 1 mGy/day and an accumulative dose of 118 mGy induced an incrementing of body weight at the 700-day but not 600-day time point. Similar irradiation of (2-month-old) Balb/c female mice with an accumulative dose of 400 mGy did not induce any weight changes at 600- and 700-day time points [53]. Tanaka et al. (2007) irradiated 8-week-old B6C3F1 mice with a low dose rate of 21 mGy/d or 1.1 mGy/day and showed a significant increase in body weight from 32 to 60 weeks of age in males and females irrespective of the fact that these mice had a significant increase in the number of multiple primary neoplasms [25]. Further study from her lab indicated that an increase in the body weight of mice with low dose rate gamma rays was due to adiposity with no corresponding increase in feed consumption [54]. A Korean team reported that mice exposed to low-dose radiation, either at very low (0.7 mGy/h) or low dose (3.95 mGy/h) rates for a total dose of 0.2 and 2 Gy, respectively, had a normal range of body weight [55]. Prenatal continuous low dose rate irradiation with 0.05, 1.0, and 20 mGy/day for 18 days did not affect offspring weight gain from 3 to 179 weeks of age. However, continuous medium dose rate irradiation with 400 mGy/day reduced male offspring weight gain from 7 to 147 weeks, while enhancing female weight gain from 15 to 87 weeks of age. From 111 to 143 weeks of age, female offspring weight declined significantly [26]. The present study showed reduced body weight, BMI and some organ weight at 1 year after prenatal irradiation. As no pathophysiological changes were observed in our study, all the reduced or increased weight changes from different research groups suggest that the sources (r-rays, ^12^C, neutrons and photons) or exposure patterns (acute or chronic) of radiation, animal gender, strain and developmental stages before or after irradiation may affect animal weight, and the body weight change may not be a sensitive or reliable marker for low dose rate radiation-induced health effects, although the sample size may also be an important factor to affect statistical analysis.

### 4.5. Summary

The present study indicates that the prenatal and postnatal (first 30 days) continuous low dose rates of irradiation with 1 or 20 mGy/d, and prenatal continuous low dose rates of irradiation with 100 mGy/d, may not induce pathological changes in different organs, in particular brain cellular distribution in the dentate gyrus of the hippocampus. SHIRPA and other neurobehavioural tests suggest that these animals may have a normal life, at least during the their first year of life. While the significant changes of miRNA and mRNA in the blood and hippocampus of animals with prenatal irradiation with 100 mGy/d remain to be further investigated, from a speculative point of view, the interaction of blood miR-181b-5p with Rubcnl may be involved in animal weight and BMI changes induced by prenatal low dose rate radiation exposure with 100 mGy/h. Our results suggest that chronic low dose rate irradiation with accumulated high doses may not be as harmful as previously expected from the linear no-threshold (LNT) model. The investigation of the health effect of chronic, low dose rates of radiation with a cumulative threshold dose may be a promising area to explore in radiobiology research, which may provide solid evidence for understanding the health effects of chronic low dose rate radiation exposure, for environmental radioprotection and safety policy preparation by the relevant authorities. The traditional LNT model can not be used for the evaluation of the health effects of chronic low dose rate radiation exposure, or guide emergency evacuation after nuclear accidents or radiation leakage with low dose rate contamination.

## Figures and Tables

**Table 1:** miRNA sequences for qRT-PCR.

miRNA Primers	Sequence
mmu-miR-68	GCTGTACTGACTTGATGAAAGTAC
mmu-miR-202-5p	GCTTCCTATGCATATACTTCTTT
mmu-miR-448-3p	TTGCATATGTAGGATGTCCCAT
mmu-miR-1298-5p	TTCATTCGGCTGTCCAGATGTA
mmu-miR-212-5p	ACCTTGGCTCTAGACTGCTTACT
mmu-miR-320-3p	AAAAGCTGGGTTGAGAGGGCGA
mmu-miR-423-5p	TGAGGGGCAGAGAGCGAGACTTT
mmu-miR-486b-5p	TCCTGTACTGAGCTGCCCCGAG
mmu-miR-486b-3p	CGGGGCAGCTCAGTACAGGA
mmu-miR-423-3p	AGCTCGGTCTGAGGCCCCTCAGT
mmu-miR-652-3p	AATGGCGCCACTAGGGTTGTG
mmu-miR-324-3p	ATT CCA CTG CCC CAG GTG CTG CT
mmu-miR-122-5p	TGGAGTGTGACAATGGTGTTTG
mmu-miR-744-5p	TGCGGGGCTAGGGCTAACAGCA
mmu-miR-181b-5p	AACATTCATTGCTGTCGGTGGGTT
mmu-let-7b-5p	TGAGGTAGTAGGTTGTGTGGTT
mmu-miR-6904-5p	TCCTGGGGTTAGAGTTGAGTGG

**Table 2:** mRNA sequences for qRT-PCR.

mRNA Primers	Sequence	Direction
Dnajb1 F	CCAATGGGTATGGGTGGCTT	forward
Dnajb1 R	GCCTTCTCCAGGGACTTTCC	reverse
Arc F	GGAGGGAGGTCTTCTACCGT	forward
Arc R	TCCTCCTCAGCGTCCACATA	reverse
Pdia4 F	GGCCTCTTGGATGTGAATGC	forward
Pdia4 R	CAGGGCTGAAAGTGTGGTGA	reverse
Fos F	AGTCAAGGCCTGGTCTGTGT	forward
Fos R	TGGAACACGCTATTGCCAGG	reverse
Tm6sf2 F	TTCTCACACATGGGTGCCTC	forward
Tm6sf2 R	CTTGGTCCTGTGGCGAAGAT	reverse
Tent5a F	CTCCAGGACTGACCAAGGC	forward
Tent5a R	CGGACACCTATGCCCTTCTC	reverse
Cdkl5 F	AACGGCGAGAATCCAAGCAT	forward
Cdkl5 R	AAGGCGTTTGTTGGTCACTGT	reverse
H2bc23 F	TACAACAAGCGCTCGACCAT	forward
H2bc23 R	TGTCACTGAACACGTGCCTT	reverse
Hspb1 F	ATAGAGACCTGAAGCACCGC	forward
Hspb1 R	CGGTCATGTTCTTGGCTGGT	reverse
Crybb3 F	AAGCAGGTCTCTGCCTCCT	forward
Crybb3 R	TACGATCTCCATCTTGCGCC	reverse
Vmn1r58 F	GGTCAAAACACGGCCAAACC	forward
Vmn1r58 R	AGGAGAAACAGCCTTCTCTCAA	reverse
Scd1 F	GAGTAGCTGAGCTTTGGGCT	forward
Scd1 R	ACTTCATCAGCGGGGACTTG	reverse
Cd59b F	CTGTTGCCTTGGATCAGCCT	forward
Cd59b R	TGATACACTTGCCTTCCGGC	reverse
Stip1 F	GTGTTCAACCAGTGAGCAGG	forward
Stip1 R	CAGGTCTGACGGCTTGTTCT	reverse
Ahsa2 F	GACCAACGTGAACAACTGGC	forward
Ahsa2 R	CGTCTTGAGTGCCTTCAGGT	reverse
Dnaja1 F	GGCTCGGCTACAAAAGAGGT	forward
Dnaja1 R	ATGCGTTCTCCATGACCCTG	reverse
Dap F	TCCCTAAAGGGTCGTTGAACC	forward
Dap R	AGGAGCCCATCCCTCCTTAG	reverse
F5 F	GCTTGCCTTCTCAAGCGTTC	forward
F5 R	CCCAAGTGACTTTGCGTGTG	reverse
Hspa1b F	GGCACCGATTACTGTCAAGG	forward
Hspa1b R	ACAGTGCCAAGACGTTTGTT	reverse
P4ha1 F	AAGACTGTTCTGCCGCTACC	forward
P4ha1 R	TTCGTAGCCAGACAGCCAAG	reverse
Rubcnl F	AGGTGATCCGAACCTGTCG	forward
Rubcnl R	TCCGAGCATCACCTACGCC	reverse
Banp F	AACACCACGAGAATTCCGCA	forward
Banp R	GCACTTTGTTGCAGGTCTGG	reverse
Abhd14b F	TAGCACACGCCATTCTCCTG	forward
Abhd14b R	AAGGTATCCACCACAGCAGC	reverse
GAPDH F	ACC ACA GTC CAT GCC ATC AC	forward
GAPDH R	TCC ACC ACC CTG TTG CTG TA	reverse

**Table 3:** Histopathological findings in male B6C3F1 mice.

Lesion	Organ	Lesion	Control (0 mGy) n = 16 (%)	1 mGy/d (48 mGy) n = 16 (%)	20 mGy/d (960 mGy) n = 16 (%)	100 mGy/d (1800 mGy) n = 16 (%)
Neoplastic lesion	Liver	Adenoma, Hepatocellular		1 (6.3)	3(18.8)	1 (6.3)
		Carcinoma, Hepatocellular	1 (6.3)	2 (12.5)		
	Lung	Adenoma, Bronchiolo-Alveolar		4 (25.0)	3 (18.8)	1 (6.3)
Non-neoplastic lesion	Adrenal gland	Accessory cortical tissue	1 (6.3)	1 (6.3)	1 (6.3)	1 (6.3)
		Hyperplasia, subcapsular cell	6 (37.5)	5 (31.3)	6 (37.5)	10 (62.5)
	Heart	Valvular endocardiosis	6 (37.5)	5 (31.3)	3 (18.8)	4 (25.0)
	Kidney	Cysts				1 (6.3)
		Hyperplasia, renal tubular		1 (6.3)		
	Liver	Cellular alteration, foci	1 (6.3)	1 (6.3)		
		Cytoplasmic vacuolization	14 (87.5)	11 (68.8)	12 (75.0)	13 (81.3)
		Granular degeneration	9 (56.3)	9 (56.3)	12 (75.0)	9 (56.3)
		Inflammation	1 (6.3)	1 (6.3)		
	Lung	Congestion	1 (6.3)	1 (6.3)	1 (6.3)	2 (12.5)
		Metaplasia, osseous			1 (6.3)	
		MNC inf, perivascular	2 (12.5)			
		Pneumonia, interstitial	1 (6.3)			2 (12.5)

## Data Availability

The data presented in this study are available on request from the corresponding author.

## Supplementary Materials

The following supporting information can be downloaded at: https://www.mdpi.com/article/10.3390/cells13171423/s1, Figure S1: mRNA and miRNA expression in the hippocampus and blood between the control and prenatal irradiated mice: Real time qPCR indicates no significant change in the expressions of F5, Dusp6, Egr2, Hspb1, CD74 in the hippocampus (Figure S1A), and of Vmn1r58, Scd1, Cd59b, Slc5a3, Ccdc117, Cish, Fosb, Gem, H2bc24, Fam122a, Sesn3 in whole blood (Figure S1B). In the blood, there is no significant change in miR 122 5p, miR 744 5p, miR let7d 3p, miR 328 3p, miR 151 3p, miR 296 5p (Figure S1C).
