# Supplementary material for: Effects of Continuous Prenatal Low Dose Rate Irradiation on Neurobehavior, Hippocampal Cellularity, Messenger RNA and MicroRNA Expression on B6C3F1 Mice
Source: Cells. 2024 Aug 26;13(17):1423. doi: 10.3390/cells13171423 (PMC11394438; doi:10.3390/cells13171423)
Supplement: Supplementary file 1 [file cells-13-01423-s001.zip › cells-3143148-supplementary.pdf]

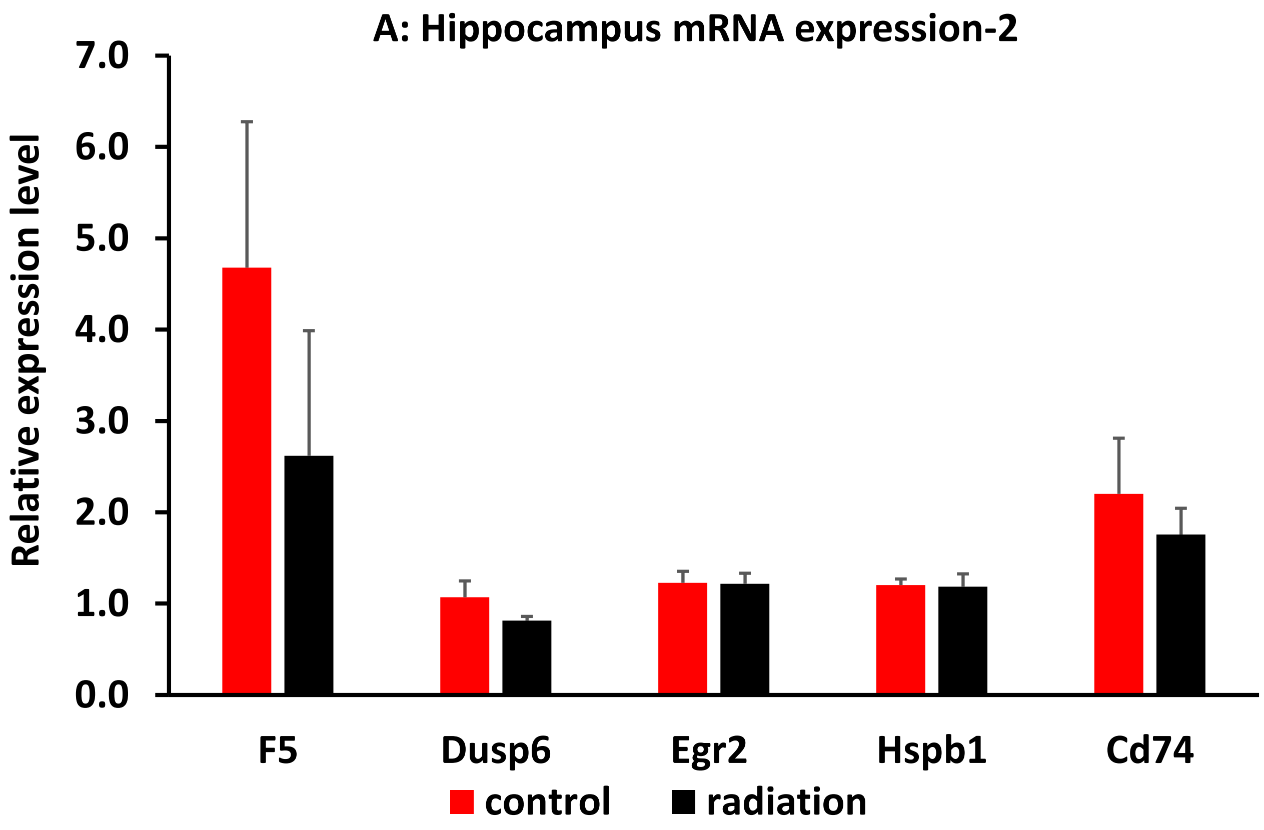
A: Hippocampus mRNA expression-2
Relative expression level
7.0
6.0
5.0
4.0
3.0
2.0
1.0
0.0
F5
Dusp6
Egr2
Hspb1
Cd74
control
radiation

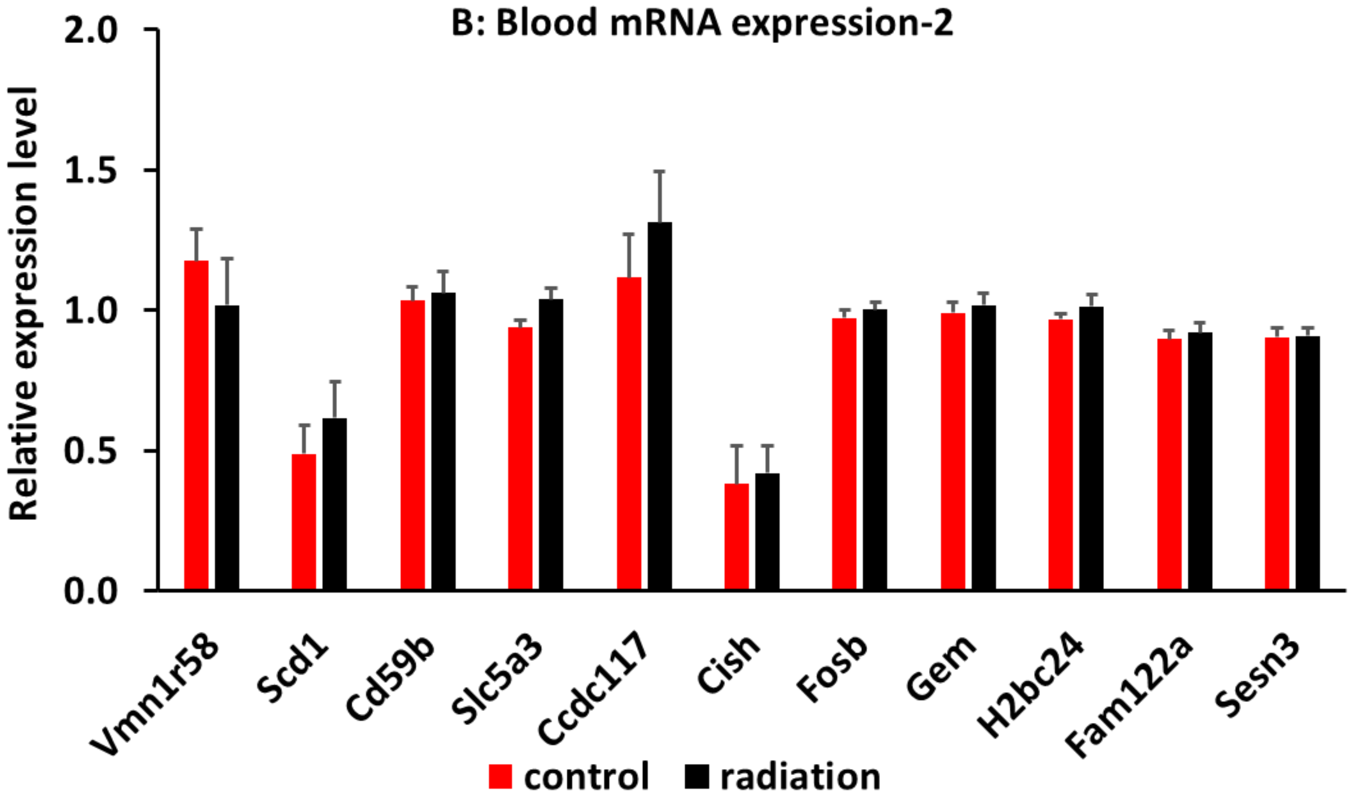
B: Blood mRNA expression-2
Relative expression level
2.0
1.5
1.0
0.5
0.0
Vmn1r58
Scd1
Cd59b
Slc5a3
Ccdc117
Cish
Fosb
Gem
H2bc24
Fam122a
Sesn3
control
radiation

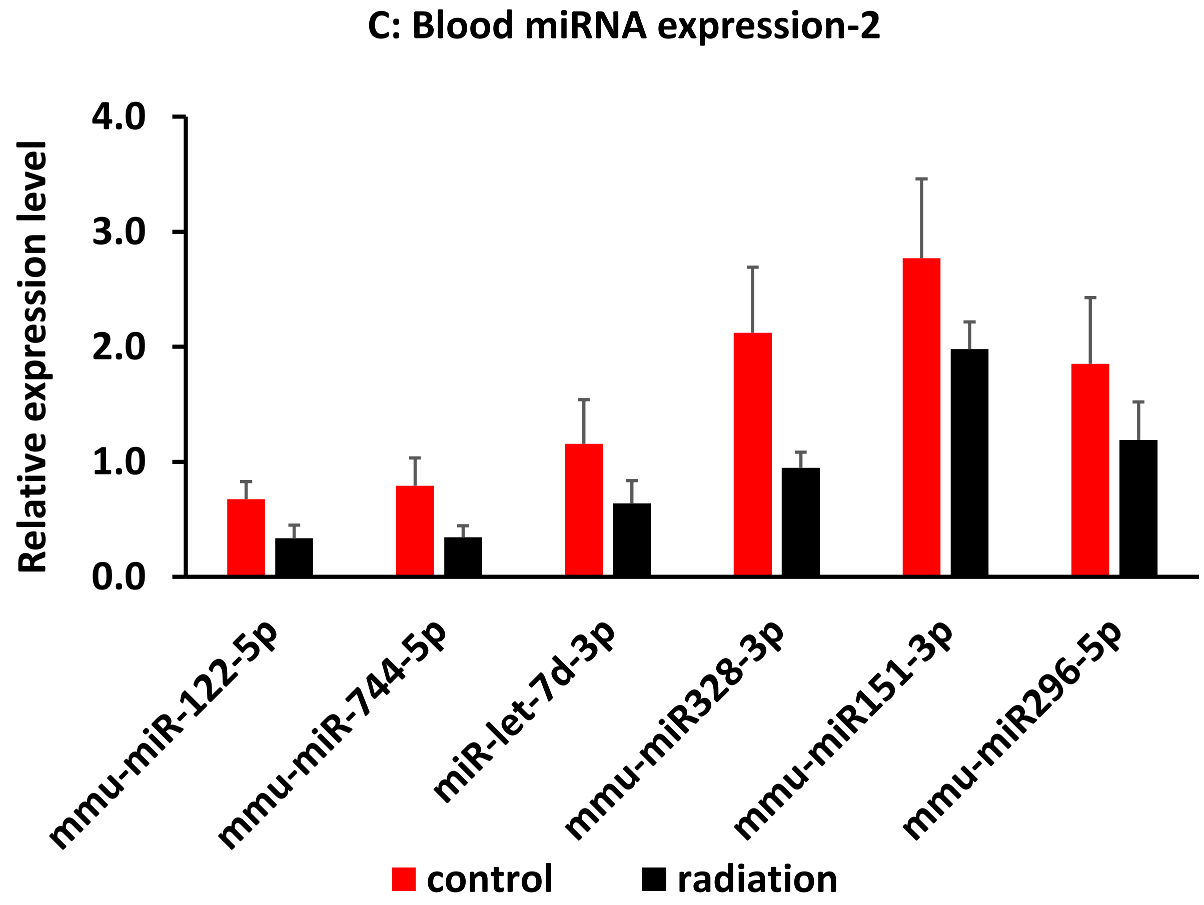
C: Blood miRNA expression-2
Relative expression level
4.0
3.0
2.0
1.0
0.0
mmu-miR-122-5p
mmu-miR-744-5p
miR-let-7d-3p
mmu-miR328-3p
mmu-miR151-3p
mmu-miR296-5p
control
radiation

Figure S1: mRNA and miRNA expression in the hippocampus and blood between the control and prenatal irradiated mice: Real-time qPCR indicates no significant change in the expressions of F5, Dusp6, Egr2, Hspb1, CD74 in the hippocampus (Fig. S1A), and of Vmn1r58, Scd1, Cd59b, Slc5a3, Ccdc117, Cish, Fosb, Gem, H2bc24, Fam122a, Sesn3 in whole blood (Fig. S1B). In the blood, there is no significant change in miR-122-5p, miR-744-5p, miR-let7d-3p, miR-328-3p, miR-151-3p, miR-296-5p (Fig. S1C)
